# Intra-Hospital, Inter-Hospital and Intercontinental Spread of ST78 MRSA From Two Neonatal Intensive Care Unit Outbreaks Established Using Whole-Genome Sequencing

**DOI:** 10.3389/fmicb.2018.01485

**Published:** 2018-07-04

**Authors:** Megan R. Earls, David C. Coleman, Gráinne I. Brennan, Tanya Fleming, Stefan Monecke, Peter Slickers, Ralf Ehricht, Anna C. Shore

**Affiliations:** ^1^Microbiology Research Unit, Dublin Dental University Hospital, Trinity College, University of Dublin, Dublin, Ireland; ^2^National MRSA Reference Laboratory, St. James's Hospital, Dublin, Ireland; ^3^Abbott (Alere Technologies GmbH), Jena, Germany; ^4^InfectGnostics Research Campus, Jena, Germany

**Keywords:** community-associated MRSA, NICU outbreak, ST78-MRSA-IVa, ST88-MRSA-IVa, whole-genome sequencing, core-genome MLST, whole-genome MLST, sequence-based plasmid analysis

## Abstract

From 2009 to 2011 [transmission period (TP) 1] and 2014 to 2017 (TP2), two outbreaks involving community-associated clonal complex (CC) 88-MRSA *spa* types t186 and t786, respectively, occurred in the Neonatal Intensive Care Unit (NICU) of an Irish hospital (H1). This study investigated the relatedness of these isolates, their relationship to other CC88 MRSA from Ireland and their likely geographic origin, using whole-genome sequencing (WGS). All 28 CC88-MRSA isolates identified at the Irish National MRSA Reference Laboratory between 2009 and 2017 were investigated including 20 H1 patient isolates, two H1 isolates recovered from a single healthcare worker (HCW) 2 years apart, three patient isolates from a second hospital (H2) and one patient isolate from each of three different hospitals (H3, H4, and H5). All isolates underwent DNA microarray profiling. Thirteen international isolates with similar microarray profiles to at least one Irish isolate were selected from an extensive global database. All isolates underwent Illumina MiSeq WGS. The majority of Irish isolates (25/28; all H1 isolates, two H2 isolates and the H3 isolate) were identified as ST78-MRSA-IVa and formed a large cluster, exhibiting 1–71 pairwise allelic differences, in a whole-genome MLST-based minimum spanning tree (MST) involving all Irish isolates. A H1/H2, H1/H3, and H1 HCW/patient isolate pair each exhibited one allelic difference. The TP2 isolates were characterised by a different *spa* type and the loss of *hsdS*. The three remaining Irish isolates (from H2, H4, and H5) were identified as ST88-MRSA-IVa and dispersed at the opposite end of the MST, exhibiting 81–211 pairwise allelic differences. Core-genome MLST and sequence-based plasmid analysis revealed the recent shared ancestry of Irish and Australian ST78-MRSA-IVa, and of Irish and French/Egyptian ST88-MRSA-IVa. This study revealed the homogeneity of isolates recovered during two NICU outbreaks (despite *spa* type and *hsdS* carriage variances), HCW involvement in the outbreak transmission chain and the strain's spread to two other Irish hospitals. The outbreak strain, CC88/ST78-MRSA-IVa, was likely imported from Australia, where it is prevalent. CC88/ST88-MRSA-IVa was also identified in Irish hospitals and was likely imported from Africa, where it is predominant, and/or a country with a large population of African descent.

## Introduction

*Staphylococcus aureus* is both a prominent pathogen worldwide and an asymptomatic coloniser of the skin and mucosal membranes of humans and animals (Young et al., 2012). The success of *S. aureus* as a disease-causing agent is largely attributable to its ability to acquire and express a wide variety of virulence-associated and antimicrobial resistance genes, many of which can be transferred horizontally between cells on mobile genetic elements (Malachowa and Deleo, 2010). *Staphylococcus aureus* develops into methicillin-resistant *S. aureus* (MRSA) upon acquisition of *mecA* or *mecC*, located on the Staphylococcal Chromosomal Cassette *mec* (SCC*mec*) mobile genetic element, or the plasmid-located *mecB* gene, all of which encode alternate penicillin-binding proteins, conferring resistance to almost all β-lactam antibiotics (Katayama et al., 2000; Shore et al., 2011; Becker et al., 2018). Molecular epidemiological evidence indicates that MRSA have evolved independently from multiple lineages of methicillin-susceptible *S. aureus* in several environments and MRSA clonal groups are therefore often categorised as healthcare-associated (HCA), community-associated (CA) or livestock-associated (LA) (Lindsay, 2010). In recent years, however, it has become increasingly apparent that clonal groups are not limited to the environment in which they initially arose (Bal et al., 2016) and thus, this type of classification currently serves to define a strain's origin.

In-depth surveillance of MRSA, both locally and internationally, is essential in order to identify modes and routes of transmission and, ultimately, to develop effective strategies to prevent or limit transmission. Whole-genome sequence analysis provides optimal typing resolution, thus enabling the accurate determination of isolate relatedness. Over the past five years, both single nucleotide polymorphism (SNP) and allele-based approaches have been utilised during the analysis of bacterial next generation sequencing data. Initially, SNP analysis alone was generally applied to data sets (Price et al., 2013). While this method provides the highest discriminatory power available, a suitable reference genome is not always available and the wide variety of SNP filters for which parameters must be set can impede inter-study comparisons (Schürch et al., 2018). This approach is therefore ideally suited to the comparison of closely related isolates using study-specific reference genomes. In 2014, Leopold et al. built on traditional multilocus sequence typing (MLST) involving just seven loci, by using all 40 finished *S. aureus* genomes available in GenBank as of June 2013 to devise a core-genome (cg)MLST scheme, consisting of 1,861 loci (Leopold et al., 2014). This method provided a standardised tool for comparing the stable core-genome of *S. aureus* isolates. The cgMLST approach is therefore particularly well-suited to determining the relatedness of isolates recovered over a relatively long period of time and/or from disparate geographic regions, when the environmental conditions to which isolates have been recently exposed and thus, the accessory genome, are of lesser relevance. Recently, extended versions of this cgMLST scheme, which include accessory genome loci, have also been employed (Roisin et al., 2016; Sabat et al., 2017). This approach is typically referred to as whole-genome (wg)MLST and is suited to local outbreak investigations, during which, the comparison of entire genomes may be both appropriate and beneficial. Although there are no definitive cgMLST or wgMLST thresholds for assigning isolate relatedness, it has been suggested that a difference of ≤24 (core genome or whole-genome) alleles may be used as an approximate clonality guideline, however, the longer the time period over which the isolates were recovered, the higher the possibility of their exceeding this threshold (Schürch et al., 2018). Furthermore, sequence-based plasmid analysis may also be used during surveillance or outbreak investigations, although significant bioinformatics expertise is required to optimise short-read plasmid assemblies (Orlek et al., 2017).

Although whole-genome sequencing (WGS) offers optimal typing resolution, conventional molecular typing methods of moderate discriminatory power, such as *S. aureus* protein A (*spa*) typing, are commonly used during hospital outbreak investigations (Frénay et al., 1996). Furthermore, for retrospective compatibility with previous studies, MRSA isolates are still assigned to a MLST clonal complex (CC) and/or sequence type (ST) (Robinson and Enright, 2004) and to one of 13 main SCC*mec* types (Shore and Coleman, 2013; Wu et al., 2015; Baig et al., 2018). Sequence types share a CC if at least five of their seven traditional MLST alleles are identical to at least one other ST in the CC (Feil et al., 2003). The application of such typing techniques also facilitates global MRSA surveillance, allowing for the broader classification MRSA strains and their general categorisation as pandemic, endemic or sporadic. For example, the CA CC88 clones, ST78-MRSA-IV, and ST88-MRSA-IV/V, have both achieved endemic status in particular geographic regions (Monecke et al., 2011). ST78-MRSA-IV is a Panton-Valentine leukocidin (PVL)-negative clone that usually harbours the antimicrobial resistance genes *blaZ* and *erm*(A) (Monecke et al., 2011). First isolated in remote Western Australia in 1995 (O'Brien et al., 2009), ST78-MRSA-IV was the fourth most prevalent clone detected in Australia in 2012, accounting for 3.6% of all MRSA and 5.1% of all CA-MRSA (Coombs et al., 2014). Reports of this clone elsewhere, however, are lacking, with the exception of a single isolate from Germany (Monecke et al., 2011). ST88 MRSA includes both PVL-positive and negative strains, often harbours the exfoliative toxin gene, *etA*, and is generally associated with SCC*mec* types IV and V, although ST88-MRSA-VI have been identified sporadically in Western Australia (Monecke et al., 2011). Although prevalent in the Far East (Ozaki et al., 2009; Zhang et al., 2009; Qiao et al., 2014) and predominant in Africa, where it accounts for 24.2–83.3% of all MRSA isolates (Breurec et al., 2011; Schaumburg et al., 2011), ST88-MRSA is sporadic in Europe and the Middle East (Monecke et al., 2012; RuŽičková et al., 2012; Vindel et al., 2014). CC88 MRSA have also been described in bulk tank milk and retail food (Parisi et al., 2016; Raji et al., 2016).

MRSA is endemic in Irish hospitals, where the HCA ST22-MRSA-IV strain has predominated since 2002 (Irish National Meticillin-Resistant *Staphylococcus aureus* Reference Laboratory, 2016). An extensive diversity of other MRSA strains and clones has also been identified in Ireland, several of which have been involved in significant hospital outbreaks, including CA strains ST772-MRSA-V, and ST1-MRSA-IV (Brennan et al., 2012; Kinnevey et al., 2014; Earls et al., 2017). Between 2009 and 2011, a suspected protracted outbreak occurred in the Neonatal Intensive Care Unit (NICU) of an Irish hospital (H1), in which seven CC88-associated MRSA-t186 isolates were identified during patient screening. Interestingly, a second suspected protracted outbreak involving a CC88-associated *spa* type occurred in the same NICU between 2014 and 2017, in which 15 MRSA-t786 screening isolates were recovered. Infants in NICUs are particularly vulnerable to serious infection and MRSA colonisation increases their risk of nosocomial infection (Geva et al., 2011). The present study used WGS to achieve three main objectives. Firstly, this study aimed to determine the relationship between isolates from both outbreaks and to identify putative transmission events. Secondly, the relatedness of the outbreak isolates to other CC88 MRSA identified in Ireland was investigated. Finally, considering that CC88 MRSA is not usually associated with Ireland or indeed other European countries, the present study sought to determine the relatedness of all CC88 MRSA recovered in Ireland to international comparator isolates.

## Materials and methods

### Isolates

All 28 CC88-MRSA isolates identified at the Irish National MRSA Reference Laboratory (NMRSARL) between January 2009 and February 2017 were investigated. Twenty-two of these isolates were recovered in the NICU of H1 during two suspected outbreaks which occurred from 2009 to 2011 (seven isolates from seven inpatients) and 2014 to 2017 (13 isolates from 13 inpatients and two isolates from one healthcare worker [HCW]), respectively. The outbreaks were initially suspected due to the number of isolates identified and their recovery in a single unit in the hospital. While this study included only one isolate per patient, two isolates (W19 and W28) were included from a single HCW, as they were recovered two years apart (in 2015 and 2017, respectively). The remaining six CC88 isolates from Ireland were recovered from inpatients of a second hospital [H2; *n* = 3 (2 NICU and one adult ward)] and in three additional hospitals (H3, *n* = 1; H4, *n* = 1; H5, *n* = 1). All Irish isolates were recovered during colonisation screening. Each isolate is represented by a letter, indicating recovery from either a patient (P) or healthcare worker (W), followed by a number, indicating the order in which the isolates were recovered (Table 1). Fifteen CC88-MRSA isolates recovered between 2001 and 2017 in Australia (*n* = 4), France (*n* = 3), Germany (*n* = 5), Tanzania (*n* = 2), and Egypt (*n* = 1) were also included in this study for comparison to the Irish isolates (Table 1). These international isolates were selected from the in-house strain collections at Abbott ([Alere Technologies GmbH] Jena, Germany) and the Institute for Medical Microbiology and Hygiene, Technical University of Dresden (Dresden, Germany) based on their genotypic similarity (*n* = 13) or dissimilarity (*n* = 2) to the Irish isolates from the present study (see DNA microarray section below for detailed description of international isolate selection). The strain collections include approximately 22,000 global *S. aureus* isolates, a selection of which have been previously reported (Monecke et al., 2008, 2011, 2016). Each international isolate is represented by a letter, indicating the country of recovery, followed by a number, indicating the order in which the isolates were recovered (Table 1).

**Table 1:** Epidemiological, phenotypic and genotypic details of the 43 CC88-MRSA isolates investigated.

**Source**	**Isolate/patient no**.	**Month and/or year of recovery**	**Sequence type^a^**	**SCC*mec* type/subtype^b^**	***spa* type^c^**	**Antimicrobial resistance profile^d^**	**Antimicrobial resistance and virulence-associated genes^e^**
Ireland-H1	P1	Jan 2009	78	IVa-MW2	t186	Ap, Er, Fx	*blaZ, cadX, erm*(A)*, mecA, hsdS, lukX, sak, scn, sec, sel*
	P2	Apr 2009	78	IVa-MW2	t186	Ap, Er, Fx	*blaZ, cadX, erm*(A)*, mecA, hsdS, lukX, sak, scn, sec, sel*
	P3	Mar 2010	78	IVa-MW2	t186	Ap, Er, Fx	*blaZ, cadX, erm*(A), *mecA, hsdS, lukX, sak, scn, sec, sel*
	P4^f^	Nov 2010	78	IVa-MW2	t186	Ap, Er, Fx	*blaZ, cadX, erm*(A)*, mecA, hsdS, lukX, sak, scn, sec, sel*
	P5^f^	Nov 2010	78	IVa-MW2	t186	Ap, Er, Fx	*blaZ, cadX, erm*(A)*, mecA, hsdS, lukX, sak, scn, sec, sel*
	P6	Aug 20/11	78	IVa-MW2	t186	Ap, Er, Fx	*blaZ, cadX, erm*(A)*, mecA, hsdS, lukX, sak, scn, sec, sel*
	P7	Sep 2011	78	IVa-MW2	t186	Ap, Er, Fx	*blaZ, cadX, erm*(A)*, mecA, hsdS, sak, scn, sec, sel*
	P10	Mar 2014	78	IVa-MW2	t786	Ap, Er, Fx	*blaZ, cadX, erm*(A)*, mecA, lukX, sak, scn, sec, sel*
	P11	Mar 2014	78	IVa-MW2	t786	Ap, Er, Fx	*blaZ, cadX, erm*(A)*, mecA, lukX, sak, scn, sec, sel*
	P13	Sep 2014	78	IVa-MW2	t786	Ap, Er, Fx	*blaZ, cadX, erm*(A)*, mecA, lukX, sak, scn, sec, sel*
	P14	Oct 2014	78	IVa-MW2	t786	Ap, Er, Fx	*blaZ, cadX, erm*(A)*, mecA, lukX, sak, scn, sec, sel*
	P15	Nov 2014	78	IVa-MW2	t786	Ap, Er, Fx	*blaZ, cadX, erm*(A)*, mecA, lukX, sak, scn, sec, sel*
	P18	Jan 2015	78	IVa-MW2	t786	Ap, Er, Fx	*blaZ, cadX, erm(A), mecA, lukX, sak, scn, sec, sel*
	W19^g^	Jan 2015	78	IVa-MW2	t786	Ap, Er, Fx	*blaZ, cadX, erm*(A)*, mecA, lukX, sak, scn, sec, sel*
	P20	June 2015	78	IVa-MW2	t786	Ap, Er, Fx	*blaZ, cadX, erm*(A)*, mecA, lukX, sak, scn, sec, sel*
	P21	Sep 2015	78	IVa-MW2	t786	Ap, Er, Fx	*blaZ, cadX, erm*(A)*, mecA, lukX, sak, scn, sec, sel*
	P22	Oct 2015	78	IVa-MW2	t786	Ap, Er, Fx	*blaZ, cadX, erm*(A)*, mecA, lukX, sak, scn, sec, sel*
	P23	Nov 2015	78	IVa-MW2	t786	Ap, Er, Fx	*blaZ, cadX, erm*(A)*, mecA, lukX, sak, scn, sec, sel*
	P24	Feb 2016	78	IVa-MW2	t786	Ap, Er, Fx	*blaZ, cadX, erm*(A)*, mecA, lukX, sak, scn, sec, sel*
	P26	June 2016	78	IVa-MW2	t786	Ap, Er, Fx	*blaZ, cadX, erm*(A)*, mecA, lukX, sak, scn, sec, sel*
	P27	June 2016	78	IVa-MW2	t786	Ap, Er, Fx	*blaZ, cadX, erm*(A)*, mecA, lukX, sak, scn, sec, sel*
	W28^g^	Feb 2017	78	IVa-MW2	t786	Ap, Er, Fx	*blaZ, cadX, erm*(A)*, mecA, lukX, sak, scn, sec, sel*
Ireland-H2	P8	Aug 2013	88	IVa- CMFT503	t786	Ap, Cm, Fx, Tp	*blaZ, cadX, cat, dfrS1, mecA, hsdS, lukX, sak, chp, scn*
	P16	Nov 2014	78	IVa-MW2	t786	Ap, Er, Fx	*blaZ, cadX, erm*(A)*, mecA, lukX, sak, scn, sec, sel*
	P17	Dec 2014	78	IVa-MW2	t786	Ap, Er, Fx	*blaZ, cadX, erm*(A)*, mecA, lukX, sak, scn, sec, sel*
Ireland-H3	P12^h^	Sep 2014	78	IVa-MW2	t786	Ap, Er, Fx	*blaZ, cadX, erm*(A)*, mecA, lukX, sak, scn, sec, sel*
Ireland-H4	P9	Sep 2013	88	IVa- CMFT503	t786	Ap, Fx, Tp	*blaZ, cadX, dfrS1, mecA, etA, hsdS, lukX, sak, chp, scn*
Ireland-H5	P25	Mar 2016	88	IVa- CMFT503	t786	Ap, Fx, Tp	*blaZ, cadX, dfrS1, mecA, etA, hsdS, lukX, sak, chp, scn*
Australia	A1	2001	78	IVa-MW2	t186	Ap, Er, Fx	*blaZ, cadX, erm*(A)*, mecA, hsdS, lukX, sak, scn*
	A2	2002	78	IVa-MW2	t186	Ap, Er, Fx	*blaZ, cadX, erm*(A)*, mecA, hsdS, lukX, sak, scn, sec, sel*
	A3	2008	78	IVa-MW2	t186	Ap, Er, Fx, Tp	*blaZ, cadX, erm*(A)*, mecA, hsdS, lukX, sak, scn, sec, sel*
	A4	2008	78	IVa-MW2	t186	Ap, Fx, Tp	*blaZ, cadX, erm*(A)*, mecA, hsdS, lukX, sak, scn, sec, sel*
Egypt	E1^i^	2014	88	IVa- CMFT503	t13712	Ap, Fx, Tp	*blaZ cadX, dfrS1, mecA, hsdS, lukX, sak, chp, scn*
France	F1	2002	88	IVa- CMFT503	t186	Ap, Er, Fx, Tp	*blaZ, cadX, dfrS1, erm*(C), *mecA, etA, hsdS, lukX, sak, chp, scn*
	F2	2002	88	IVa- CMFT503	t786	Ap, Er, Fx, Tp	*blaZ, cadX, dfrS1, erm*(C), *mecA, etA, hsdS, lukX, sak, chp, scn*
	F3	2002	88	IVa- CMFT503	t690	Ap, Fx, Te, Tp	*blaZ, cadX, dfrS1, mecA, tet*(K), *vga*(A), *etA, hsdS, lukX, sak, chp, scn*
Germany	G1	2008	78	IVa-MW2	t186	Ap, Er, Fx	*blaZ, cadX, erm*(A)*, mecA, hsdS, lukX, sak, scn*
	G2	2016	88	IVa- CMFT503	t1028	Ap, Fx, Te, Tp	*blaZ, cadX, dfrS1, mecA, tet*(K), *hsdS, lukX, sak, chp, scn*
	G3	2017	88	IVa- CMFT503	t786	Ap, Fx, Tp	*blaZ, cadX, dfrS1, mecA, hsdS, lukX, sak, chp, scn*
	R1	2014	88	IVa-MW2	t17863	Ap, Er, Fx	*blaZ, cadX, erm*(C), *mecA, hsdS, lukF, lukS, lukX, sak, chp, scn*
	R2	2017	88	IVa-MW2	t5041	Ap, Fx, Te, Cp	*blaZ, cadX, mecA, tet*(K), *hsdS, lukF, lukS, lukX, sak, chp, scn*
Tanzania	T1	2016	88	IVa- CMFT503	t690	Ap, Fx, Tp	*blaZ, cadX, dfrS1, mecA, hsdS, lukX, sak, chp, scn*
	T2	2016	88	IVa- CMFT503	t690	Ap, Fx, Tp	*blaZ, cadX, dfrS1, mecA, vga*(A), *etA, hsdS, lukX, sak, chp, scn*

a Sequence types (STs) were assigned using Ridom SeqSphere+ version 4.1 (Ridom GmbH, Germany). Allelic profiles: ST78, 22-1-14-23-12-53-31; ST88, 22-1-14-23-12-4-31.

b All SCCmec subtypes were detected using an SCCmec subtyping DNA microarray (Monecke et al., 2016). All Irish isolates underwent in silico analysis for predicted DNA SCCmec subtype microarray hybridisation profiles, while all other isolates underwent real-life DNA microarray analysis. Both SCCmec subtypes IVa-MW2 (GenBank accession: BA000033.2) and IVa-CMFT503 (GenBank accession: HF569113.1) have been described previously (Monecke et al., 2016).

c All H1 t186 isolates were involved in an outbreak between 2009 and 2011. All H1 t786 isolates were involved in an outbreak between 2014 and 2017. spa repeat successions: t186, 07-12-21-17-13-13-34-34-33-34; t786, 07-12-21-17-13-34-34-33-34; t690, 07-12-21-17-13-13-34-34-34-33-34; t1028, 07-34-33-34; t5041, 07-12-21-17-13-13-34-34-34-34-33-34; t13712, 07-12-21-17-13; t17863, 07-12-12-13-13-13-34-33-34.

d Antimicrobial resistance phenotypes were determined by testing the susceptibility of isolates to a panel of 20 antimicrobial agents including amikacin, ampicillin (Ap), cefoxitin (Fx), chloramphenicol (Cm), ciprofloxacin (Cp), clindamycin, erythromycin (Er), fusidic acid, gentamicin, kanamycin, linezolid, mupirocin, neomycin, rifampicin, streptomycin, sulphonamide, tetracycline, tobramycin, trimethoprim (Tp) and vancomycin.

e All antimicrobial resistance and virulence-associated genes were detected using the S. aureus Genotyping Kit 2.0 system [Abbott (Alere Technologies GmbH), Jena, Germany]. All Irish isolates underwent in-silico analysis for predicted Genotyping Kit 2.0 DNA microarray hybridisation profiles, while all other isolates underwent real-life DNA microarray analysis.

f Isolates P4 and P5 were recovered from twins on the same day.

g Isolates W19 and W28 were recovered from the same healthcare worker two years apart.

h The patient from whom isolate P12 was recovered had been transferred from H1.

i Isolate E1 was recovered from a buffalo. All other isolates were recovered from humans.

*H, hospital; NA, not available*.

Isolates were identified as *S. aureus* using the tube coagulase test or the Vitek MS Matrix-Assisted Laser Desorption Ionization-Time of Flight Mass Spectrometry system (Vitek, bioMérieux, Marcy l'Etoile, France) according to the manufacturer's instructions. Methicillin resistance was detected using 30-μg cefoxitin disks (Oxoid Ltd., Basingstoke, United Kingdom) in accordance with European Committee of Antimicrobial Susceptibility Testing methodology and interpretive criteria (European Committee on Antimicrobial Susceptibility Testing, 2017) or using the automated VITEK 1 or VITEK 2 systems (bioMérieux, Nuertingen, Germany). Isolates were stored at −80°C on individual Protect Bacterial Preservation System cryogenic beads (Technical Services Consultants Ltd., Heywood, United Kingdom).

### Phenotypic susceptibility testing

The susceptibility of all isolates was determined against a panel of 19 antimicrobial agents, in addition to cefoxitin, by disk diffusion using European Committee of Antimicrobial Susceptibility Testing methodology (European Committee on Antimicrobial Susceptibility Testing, 2017), and previously described reference strains and interpretative criteria (McManus et al., 2015). The 19 agents tested were amikacin, ampicillin, chloramphenicol, clindamycin, ciprofloxacin, erythromycin, fusidic acid, gentamicin, kanamycin, linezolid, mupirocin, neomycin, rifampicin, streptomycin, sulphonamide, tetracycline, tobramycin, trimethoprim, and vancomycin.

### *spa* typing

Genomic DNA for *spa* typing was extracted using the 6% InstaGene matrix solution, according to the manufacturer's instructions (BioRad, München, Germany). The variable X region in the *spa* gene of each isolate underwent PCR amplification using the primers and thermal cycling conditions described by the European Network of Laboratories for Sequence Based Typing of Microbial Pathogens (SeqNet; http://www.seqnet.org). Resulting PCR products were purified using the GenElute PCR clean-up kit (Sigma-Aldrich Ireland Ltd., Wicklow, Ireland) and were sequenced commercially (Source Bioscience, Waterford, Ireland) using an ABI 3730xl Sanger sequencing platform. The Ridom StaphType software package version 1.5 (Ridom Gmbh, Würzburg, Germany) was used for *spa* sequence analysis and *spa* type assignment.

### Genotyping and SCC*mec* subtyping using DNA microarrays

The 15 international CC88 isolates underwent DNA microarray profiling using the *S. aureus* Genotyping Kit 2.0 system [Abbott (Alere Technologies GmbH)] and an additional SCC*mec* typing array (Monecke et al., 2016). The *S. aureus* Genotyping Kit 2.0 DNA microarray detects 333 target sequences, corresponding to approximately 170 different genes and their allelic variants, and encoding antimicrobial resistance and virulence-associated genes, species, and typing markers, and several SCC-associated marker genes. It assigns *S. aureus* isolates to MLST CCs/STs and SCC*mec* types. Detailed descriptions of the relevant genes, primers, and probes have been previously described (Monecke et al., 2008). The SCC*mec* typing array targets an additional 83 distinct sequences that are variably present in the SCC*mec* element and which form the basis of a previously described system that distinguishes between 61 different SCC*mec* subtypes (Monecke et al., 2016). Detailed descriptions of these SCC*mec*-linked genes/alleles and their corresponding primers and probes have been previously published (Monecke et al., 2016). Genomic DNA was extracted for both DNA microarray profiling and SCC*mec* array subtyping by enzymatic lysis using the *S. aureus* Genotyping Kit 2.0 [Abbott (Alere Technologies GmbH)] and the Qiagen DNeasy blood and tissue kit (Qiagen, West Sussex, United Kingdom). As all Irish isolates underwent WGS as part of the present study (see below), their genome sequences underwent *in silico S. aureus* Genotyping Kit 2.0 microarray profiling and SCC*mec* array subtyping. Virtual DNA array hybridisation patterns were generated whereby contigs were searched for probe binding sites and signal strength was dictated by the number of nucleotide mismatches, as previously described (Monecke et al., 2016). The CC, DNA microarray profile and SCC*mec* type/subtype was determined for each Irish isolate. Thirteen international isolates, with a similar DNA microarray profiles to at least one Irish isolate, were selected from the aforementioned global database, for WGS (Table 1). Two international “reference isolates” with dissimilar DNA microarray profiles to any of the Irish isolates were also selected for WGS, as controls (Table 1).

### Whole-genome sequencing

All isolates underwent WGS using genomic DNA extracted as described above for DNA microarray profiling. DNA quality was assessed by UV absorbance using the NanoDrop spectrophotometer 2000 (ThermoFisher Scientific, Dublin, Ireland) and dilutions were performed using the Qubit Fluorometer 3.0 (ThermoFisher Scientific). The Nextera XT DNA Library Preparation Kit (Illumina, Eindhoven, The Netherlands) was used according to the manufacturer's instructions and libraries underwent paired-end sequencing using the 500-cycle MiSeq Reagent Kit v2 (Illumina). Libraries were scaled to exhibit at least 100x coverage and the quality of each sequencing run was assured following cluster density and Q30 assessment.

### Whole-genome sequence analysis

#### WgMLST and cgMLST

WGS data were analysed using the wgMLST scheme available in BioNumerics v7.6 (Applied Maths, Sint-Martens-Latem, Belgium) consisting of 3,904 *S. aureus* wgMLST loci (Roisin et al., 2016), including 1,861 cgMLST loci (Leopold et al., 2014). In order to ensure that all relevant alleles present were detected, two separate algorithms were used to generate a consensus whole-genome MLST profile for each isolate. The first method determined locus presence/absence and allelic identity using an assembly-free k-mer approach. The second, assembly-based method, used a BLAST approach to detect alleles on contigs assembled using the SPAdes software v3.7.1 (Bankevich et al., 2012) incorporated into BioNumerics. Default base correction parameters were applied and all contigs below 1000 bp were removed. The default settings were used for both the assembly-free and assembly-based algorithms. The quality of the sequence read sets, *de novo* assemblies, and assembly-free, and assembly-based allele calls, were assessed using the quality statistics window in BioNumerics and are detailed in Dataset S1. Traditional MLST sequence types were assigned using Ridom SeqSphere+ version 4.1 (Ridom GmbH, Germany).

#### SNP analysis

Isolates confirmed to be closely related following wgMLST subsequently underwent SNP analysis using a study-specific reference sequence. The SPAdes assembly of isolate P6 was chosen as the reference sequence due to both its central position in the wgMLST-based minimum spanning tree (MST) cluster and the high quality of its assembly. The BioNumerics Power Assembler mapping algorithm was used to create a consensus sequence for each sample and a pairwise distance matrix was generated. SNPs were called exclusively in positions shared by all samples. Only SNPs with at least 40x coverage were considered. Potentially indel-related SNPs, occurring within 12 bp of each other, were removed. Positions with ambiguous base calls and SNPs in repetitive regions were excluded.

#### Minimum spanning trees

Minimum spanning trees were constructed firstly, involving the Irish isolates exclusively and secondly, involving both the Irish and international isolates. For the Irish isolates, in order to identify the most appropriate analysis method, three separate MSTs were generated based on cgMLST, wgMLST or SNP data, and were examined in tandem with all available epidemiological and genotypic information. As the Irish and international isolates were recovered over 16 years and from disparate geographic regions, the construction of a cgMLST-based MST was deemed appropriate in this instance. All MSTs were generated using the permutation resampling function and default priority rule set in BioNumerics. The resampling support for each branch was examined to ensure the validity of the general MST structure.

### Sequence-based plasmid analysis

All isolate genomes underwent sequence-based plasmid analysis. Sequence read sets were assembled using SPAdes v3.11.1 (Bankevich et al., 2012) with a final kmer size of 127. All contigs under 500 bp and all conitgs with kmer coverage less than 3.0 were excluded. For each isolate, a scatter plot was generated depicting the GC content versus coverage for each contig. Putative plasmid-derived contigs were differentiated from chromosomal-derived contigs based on their elevated coverage, low GC content and the identity of their first and last 127 nucleotides, indicative of a circular replicon. All putative plasmid-derived contigs were blasted against The National Centre for Biotechnology Information database (https://blast.ncbi.nlm.nih.gov/Blast.cgi) and those which mapped to a known plasmid sequence in GenBank were considered to be confirmed plasmids. Any plasmid types present in the Irish isolates or in both the Irish and international isolates were identified. For each such plasmid type, a multi-sequence global alignment was constructed including all the newly identified plasmid sequences and the GenBank reference sequence, using MAFT v7.273 (Katoh et al., 2002).

## Results

### Two distinct clusters of CC88-MRSA isolates

The majority of Irish isolates were identified as *spa* type t786 (*n* = 21), while those remaining were identified as t186 (*n* = 7; Table 1). All seven t186 isolates were recovered during the first suspected H1 outbreak, while the t786 isolates were recovered either during the second suspected H1 outbreak (*n* = 13) or from one of the four alternative hospitals (H2-H5; Table 1). The majority of Irish isolates (25/28) were also identified as ST78-MRSA-IVa, harbouring an SCC*mec* type IVa element corresponding to that identified in the MW2 MRSA strain (GenBank accession: BA000033.2). This included all t186 isolates (7/7) and 18/21 t786 isolates. The remaining three t786 isolates were assigned to ST88, a single locus variant of ST78, and harboured an SCC*mec* type IVa element corresponding to that identified in the CMFT503 MRSA strain (GenBank accession: HF569113.1; Table 1). The presence of a hypothetical SCC*mec* terminus protein, Q9XB68, in the MW2-like SCC*mec* element and the presence of both an alternate SCC*mec* terminus protein, SCC*mec* terminus 01, and the LytTR domain DNA-binding regulator, Q931B7, in the CMFT503-like SCC*mec* element, distinguish the two SCC*mec* type IVa elements (Monecke et al., 2016).

**Figure 1:**
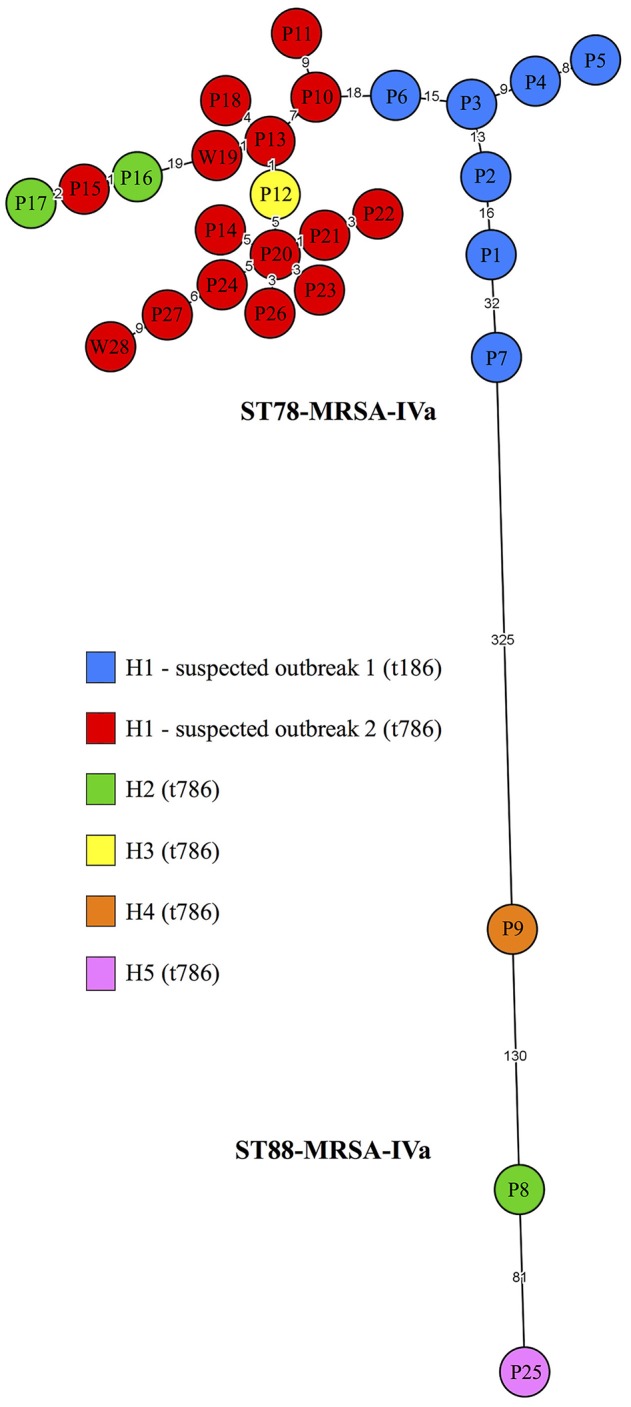
A minimum spanning tree based on whole-genome multilocus sequence typing profiles of 28 CC88-MRSA isolates recovered in Irish hospitals between 2009 and 2017. Isolates are numbered in the order in which they were recovered. Isolate *spa* types are indicated by the colour legend. Suspected outbreak 1, involving seven t186 isolates, occurred in the NICU of H1 between 2009 and 2011. Suspected outbreak 2, involving 15 t786 isolates, occurred in the same NICU between 2014 and 2017. While one isolate was included per patient, two isolates (W19 and W28) recovered two years apart were included from a single healthcare worker. The remaining isolates were recovered from four different Irish hospitals. Isolates were identified as either ST78-MRSA-IVa or ST88-MRSA-IVa. Branch labels represent allelic distances. H, hospital; NICU, neonatal intensive care unit.

Interestingly, a highly conserved gene within *S. aureus* lineages, the type I restriction modification system gene, *hsdS* (Waldron and Lindsay, 2006), was absent from all t786 ST78-MRSA-IVa isolates. It was subsequently noted that, according to the cgMLST-based MST, this unusual deletion had occurred twice within a relatively small population (Figure S1). Importantly, however, the wgMLST-based MST (Figure 1) indicated that the *hsdS* deletion occurred just once within this population. It was therefore concluded that the wgMLST-based tree likely depicted the evolutionary path of this strain more accurately than the cgMLST tree. To confirm/dispute this finding, a SNP-based MST was generated involving the relevant isolates (Figure S2). The structure of this tree was in agreement with that of the wgMLST MST, confirming that the *hsdS* deletion likely occurred once during the strain's spread. Ultimately, considering that the application of SNP analysis was not appropriate for all 28 Irish isolates, the wgMLST-based MST was selected for detailed data interpretation. Therefore, any allelic distances stated herein between Irish isolates exclusively, refer to wgMLST loci, while those stated between Irish and international isolates, or between international isolates exclusively, refer to cgMLST loci, as outlined in the methods.

Whole-genome MLST further supported the differentiation of isolates as suggested by their SCC*mec* subtypes and traditional STs, grouping all 25 ST78-MRSA-IVa isolates into a large cluster at one end of a MST, while the three ST88-MRSA-IVa isolates dispersed at the opposite end of the tree. The ST78-MRSA-IVa and ST88-MRSA-IVa isolates differed by a minimum of 325 alleles and exhibited average pairwise allelic distances of 23.8 (min. = 1; max. = 71) and 140.7 (min. = 81; max. = 211), respectively (Figure 1).

#### ST78-MRSA-IVa

The 25 ST78-MRSA-IVa isolates which grouped into the large wgMLST-MST cluster were recovered at intervals of 0–30 months and included all H1 isolates, two H2 isolates (P16 and P17) and the H3 isolate (P12). All patients from whom ST78-MRSA-IVa isolates were recovered were neonates. The patient from whom the H3 isolate was recovered, had recently been transferred from H1. All ST78-MRSA-IVa isolates exhibited resistance to both ampicillin and erythromycin, and harboured the β-lactamase resistance gene, *blaZ*, the macrolide-lincosamide, and streptogramin B resistance gene, *erm*(A), the cadmium tolerance gene, *cadX*, the immune evasion complex (IEC) genes *sak*, and *scn* (IEC type E) and the enterotoxin genes *sec*, and *sel* (Table 1). Isolate P7 was the only isolate that lacked the leukocidin homologue, *lukX*. The maximum distance observed between any two directly linked nodes was 32 alleles, detected between t186 isolates, P1 and P7, which were recovered almost 3 years apart during suspected outbreak 1. All other directly linked isolates exhibited 1–19 allelic differences, significantly fewer than the recently proposed approximate clonality threshold of 24 alleles. This indicated a high degree of relatedness between the vast majority of directly linked isolates and a significant relationship between all isolates within the cluster network (Figure 1). Furthermore, there were no apparent sub-clusters dictated by *spa* type, suggesting that the “two outbreak strains” were homogeneous. Specifically, the branch that linked isolates P6 (t186) and P10 (t786) constituted the only direct link between the t786 and t186 isolates. However, this branch represented an allelic distance of 18, lower than both those of 19 and 32, each of which was observed elsewhere in the MST cluster. Interestingly, while the largely linear structure of the t186 isolates indicated that the outbreak strain was transmitted in a relatively sequential manner between 2009 and 2011, the highly branched network of t786 isolates suggested that a more complex transmission chain was established between 2014 and 2017 (Figure 1).

Isolates P4 and P5, which exhibited eight allelic differences, were recovered from twins on the same day, suggesting that parallel or sequential acquisition may have occurred in this instance (Figure 1). One of the H2 isolates, P16, and a H1 isolate (P15) recovered 6 days before isolate P16, exhibited one allelic difference, strongly indicating that the outbreak strain spread between these two hospitals and suggesting transmission from the same source (Figure 1). Isolate P16 and the second H2 isolate (P17), which was recovered 44 days after isolate P16, exhibited three allelic differences, indicating further spread of this strain in H2 (Figure 1). Similarly, the only H3 isolate (P12) and a H1 isolate (P13) recovered 4 days after the H3 isolate, exhibited one allelic difference, clearly indicating that the outbreak strain spread from H1 to H3, and suggesting transmission from the same source (Figure 1). Interestingly, the two t786 isolates (W19 and W28) recovered from the same HCW two years apart exhibited 20 allelic differences, indicating that the strain had either altered over time *in vivo*, or that the HCW transiently carried different variants of the strain. Isolates W19 and W28 differed from the other t786 isolates by 1–21 (average:10.5) and 9–37 (average: 21.3) alleles, respectively, suggesting transmission of the outbreak strain between patients and the HCW (Figure 1).

#### ST88-MRSA-IVa

The three ST88-MRSA-IVa isolates, which exhibited an average of 140.7 pairwise allelic differences, included the final H2 isolate (P8), and the H4 (P9), and H5 (P25) isolates, all of which were t786 (Table 1 and Figure 1). None of the patients from whom ST88-MRSA-IVa isolates were recovered were neonates (patients were aged 15 months, 25 years and 66 years). Two of the ST88-MRSA isolates (P8 and P9) were recovered from patients with names suggestive of a family connection to an African country. The phenotypic resistance profiles varied slightly amongst the ST88-MRSA-IVa isolates, all of which exhibited resistance to both ampicillin and trimethoprim, while isolate P8 exhibited chloramphenicol resistance (Table 1). The ST88-MRSA-IVa isolates also exhibited slightly differing genotypic profiles, all harbouring resistance genes *dfrSI*, encoding trimethoprim resistance*, blaZ*, and *cadX*, and the IEC genes *chp, sak*, and *scn* (IEC type B), while isolate P8 carried the chloramphenicol resistance gene, *cat*, and isolates P9 and P25 harboured *etA*, encoding exfoliative toxin A (Table 1). Considering these differences, the lack of epidemiological links and most importantly, the number of alleles by which they differed, these three isolates did not appear to be closely related.

### Relatedness of irish and international CC88 MRSA

Five of the 13 international CC88-MRSA isolates exhibiting similar array profiles to the Irish isolates were identified as ST78-MRSA-IVa-MW2. This included one German isolate (G1) recovered in 2008 and four Australian isolates, A1-A4, recovered in 2001, 2002, 2008 and 2008, respectively (Table 1). The remaining eight international isolates exhibiting similar array profiles to the Irish isolates were identified as ST88-MRSA-IVa-CMFT503. This included three French isolates (F1-3) recovered in 2002, two Tanzanian isolates (T1 and T2) recovered in 2016, one Egyptian isolate recovered in 2014 (E1) and two German isolates, G4 and G5, recovered in 2016 and 2017, respectively (Table 1). The two international reference isolates (R1 and R2 recovered in Germany in 2014 and 2017, respectively), were identified as ST88-MRSA-IVa-MW2. Following the construction of a cgMLST-based MST including all Irish and international isolates (Figure S3), international isolates R1 and R2 were excluded from further analysis as they failed to cluster with any other isolates, differing from their (shared) most closely related isolate (A1) by 189 and 198 alleles, respectively.

#### Irish and international ST78-MRSA-IVa isolates

**Figure 2:**
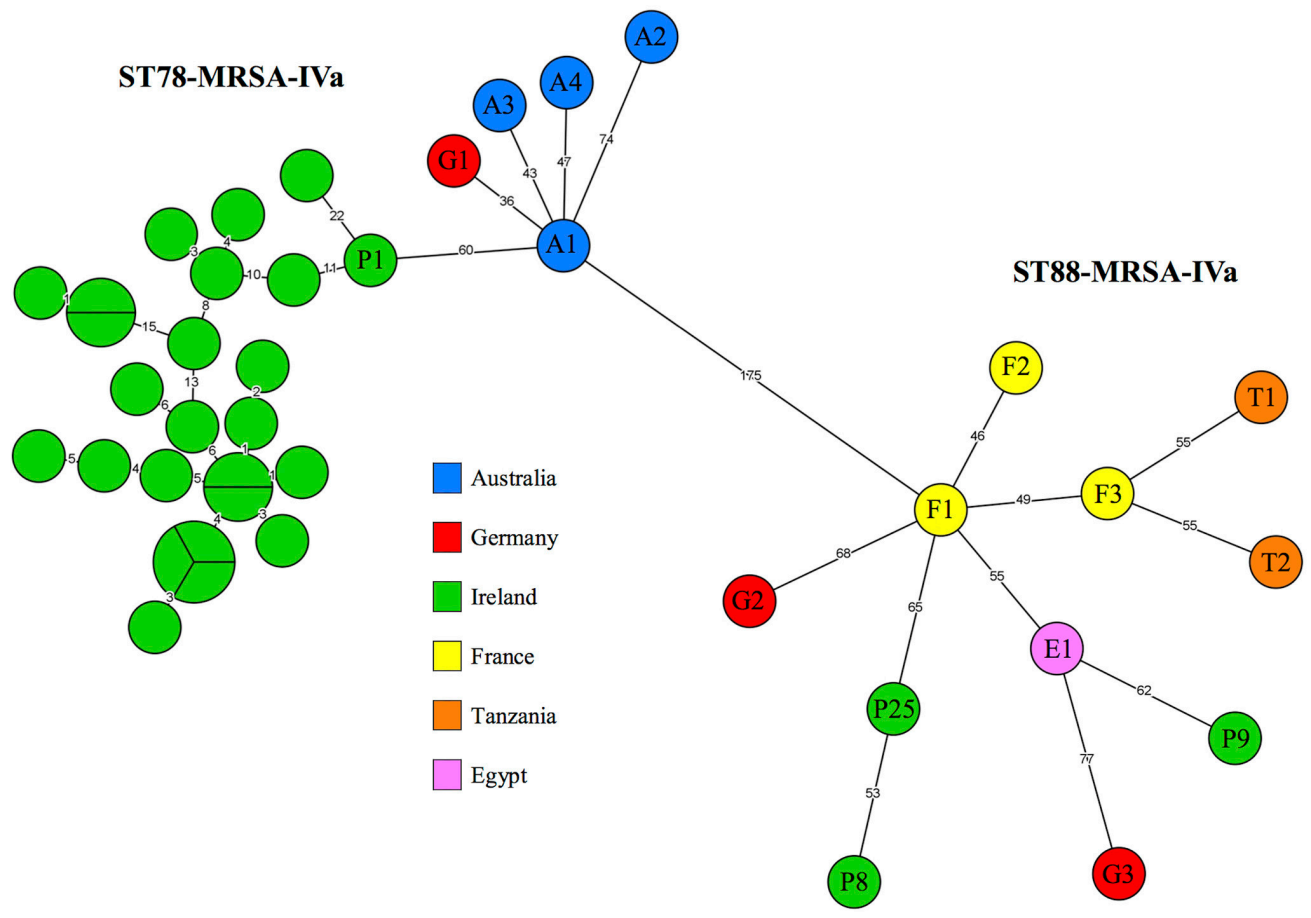
A minimum spanning tree based on core-genome multilocus sequence typing profiles of 28 Irish and 13 international CC88-MRSA isolates recovered between 2001 and 2017. The countries in which the isolates were recovered are indicated by the colour legend. Isolates were identified as either ST78-MRSA-IVa or ST88-MRSA-IVa. Branch labels represent allelic distances.

The five international ST78-MRSA-IVa isolates harboured the same resistance and virulence-associated genes as the Irish ST78-MRSA-IVa isolates (Table 1). The Irish and international ST78-MRSA-IVa isolates also exhibited very similar phenotypic susceptibility profiles, with both isolate groups exhibiting ampicillin and erythromycin resistance, while two international isolates (A3 and A4) also exhibited trimethoprim resistance. Interestingly, all international ST78-MRSA-IVa isolates were identified as t186 and none exhibited the *hsdS* deletion that characterised the Irish t786 ST78-MRSA-IVa isolates. Following the generation of a cgMLST-based MST including Irish and international isolates (Figure 2), the international ST78-MRSA-IVa isolates grouped in relatively close proximity to the Irish ST78-MRSA-IVa cluster and exhibited an average pairwise allelic distance of 68.7 (min. = 36; max. = 105; Figure 2). Specifically, isolates A2-A4 and G1 all radiated independently from isolate A1, which was the only isolate that linked directly to the t186 side of the Irish cluster (Figure 2). Isolate A1 (recovered in 2001 in Australia) and its most closely related Irish isolate (P1; recovered in 2009) exhibited 60 allelic differences which, considering the disparate geographic regions and different time periods in which they were recovered, suggested a significant degree of relatedness between these two isolates.

All Irish and international ST78-MRSA-IVa isolates harboured a 21 kb plasmid encoding *blaZ* and *cadX*, corresponding to plasmid pWBG763 (GenBank accession number: GQ900467.1). Interestingly, however, the Irish ST78-MRSA-IVa isolates were characterised by a 100 bp deletion in this plasmid. All Irish and two Australian (A2 and A3) ST78-MRSA-IVa isolates also harboured a cryptic 2 kb plasmid corresponding to pWBG764 (GenBank accession number: GQ900468.1). Both plasmids pWBG763 and pWBG764 were originally sequenced from the same ST78-MRSA-IVa strain (WBG8366), which was recovered in remote Western-Australia in 1995.

#### Irish and international ST88-MRSA-IVa isolates

The eight international ST88-MRSA-IVa isolates harboured similar resistance and virulence-associated genes to the Irish ST88-MRSA-IVa, all carrying *blaZ, cadX, dfrS1*, and the IEC genes *chp, sak* and *scn* (IEC type B), while exhibiting variable *etA* carriage (Table 1). However, two of the international ST88-MRSA-IVa isolates (F1 and F2) also harboured *erm*(C), encoding macrolide resistance, while two others (F3 and G2) harboured *tet*(K), encoding tetracycline resistance and a third pair (F3 and T2) harboured *vga*(A), encoding streptogramin A resistance, none of which were present in the Irish isolates (Table 1). The Irish and international ST88-MRSA-IVa isolates also exhibited similar phenotypic susceptibility profiles, with both isolate groups exhibiting ampicillin and trimethoprim resistance, while two international ST88-MRSA-IVa isolates (F1 and F2) exhibited erythromycin resistance and two others (F3 and G2) exhibited tetracycline resistance, neither of which were observed in the Irish ST88-MRSA-IVa isolates. None of the international ST88-MRSA-IVa isolates exhibited chloramphenicol resistance, which was observed in one Irish ST88-MRSA-IVa isolate (P8). Isolate F1 was identified as t186, while the remaining international ST88-MRSA-IVa isolates were identified as t13712 (*n* = 1), t1786 (*n* = 2), t690 (*n* = 3) or t1028 (*n* = 1; Table 1). The eight international ST88-MRSA-IVa isolates formed a dispersed cluster with the three Irish ST88-MRSA-IVa isolates, in which an average pairwise allelic distance of 78.6 was observed (min. = 46; max. = 114; Figure 2). Irish isolates P25 and P9 differed from their most closely related international isolates (F1 and E1, respectively) by 65 and 62 alleles, respectively, indicative of shared ancestral genotypes. Irish isolate P8 and its most closely related international isolate (F1) exhibited 188 allelic differences, suggesting a lack of relatedness between the two isolates (Figure 2).

All Irish and international ST88-MRSA-IVa isolates harboured a 25 kb plasmid encoding *blaZ* and *cadX*, which corresponded to contig 10 (GenBank accession number: FMNJ01000010.1) of a previously published WGS project involving an MRSA isolate (GenBank accession number: FMNJ01000000.1) recovered in Tanzania in 2008. Irish ST88-MRSA-IVa isolate P8 also harboured a *cat*-encoding plasmid, which corresponded to contig 32 (GenBank accession number: LFNS01000032.1) of a previously published WGS project involving an ST3019 *S. aureus* isolate recovered in Ghana in 2013 (GenBank accession number: LFNS00000000.1).

## Discussion

The present study revealed the homogeneity of isolates involved in two outbreaks in the NICU of an Irish hospital. Although isolate *spa* types and recovery dates suggested that two different CC88-MRSA strains may have been involved in the outbreaks in this NICU, wgMLST revealed that these outbreaks were caused by the same CC88/ST78-MRSA-IVa strain, which spread within the ward during two separate transmission periods (transmission periods 1 and 2). This investigation highlighted both the involvement of a HCW in the outbreak transmission chain and the strain's spread to two other Irish hospitals. A cgMLST-based comparison with international comparator isolates revealed that the outbreak strain was most likely imported from Australia, where it is among the prevalent MRSA clones. This study also identified a second CC88-MRSA clone present in Irish hospitals, ST88-MRSA-IVa, which was likely imported from Africa, where it is predominant, and/or a country with a large population of African ethnic origin.

Transmission period 1 (TP1) involved the intermittent acquisition of the outbreak strain by seven NICU patients over a 32-month period. Interestingly, the topology of the MST indicated that all TP1 isolates, apart from P7, were acquired in a relatively sequential chain of transmission. While the topological characteristics of a phylogenetic tree can reveal invaluable relatedness and transmission details, both previously published studies and epidemiological data must also be drawn upon in order to gain meaningful insights into the dynamics of an outbreak. Notably, previous studies have indicated that patient-to-patient transmission is rare in adult intensive care units (Price et al., 2014; Wesley Long et al., 2014), a finding which is likely applicable to NICUs given the dependency of neonates on adults for mobility. Furthermore, it is unlikely that patients P1-P6 had overlapping stays (excluding twins, P4, and P5), considering the dates on which their isolates were recovered (Table 1). It may therefore be concluded that patient-to-patient transmission did not play a significant role in the outbreak during this period. Similarly, given that previous research suggests that *S. aureus* can survive a maximum of 90 days on hospital plastics and fabrics (Neely and Maley, 2000), and isolates P1-P6 were recovered at intervals of 4–11 months (Table 1), it is perhaps unlikely that patients P1-P6 acquired the outbreak strain directly from their environment without the involvement of another intermediary factor(s). Finally, previous studies have identified a role for HCWs in the transmission of MRSA to NICU patients (Geva et al., 2011; Brennan et al., 2012; Azarian et al., 2016). Considering these points, it appears highly likely that HCWs were involved in the spread of the outbreak strain during TP1, however, as routine HCW screening did not occur, their exact role cannot be definitively determined. Furthermore, these considerations, in combination with the topology of the MST, indicate that more than one vector was involved in spreading the outbreak strain during TP1. The data suggest two possible scenarios. Firstly, it is possible that a HCW constituted the primary outbreak source, originally seeding the outbreak strain in 2009, transmitting it to patient P1, and again in 2011, transmitting it to patient P7, while a different HCW initiated the strain's spread to patients P2-P6 (Table 1; Figure 1). Alternatively, the data suggest that patient P1 constituted the primary outbreak source and, while one HCW initiated transmission to patients P2-P6, a different HCW, who had also acquired the strain in 2009, eventually transmitted it to patient P7.

In Ireland, neonates are generally screened for MRSA upon admission into a NICU and weekly, thereafter (Irish Department of Health, 2013). Interestingly, however, the outbreak strain identified here was not detected between 2011 and 2014. It is unknown whether any staff changes or staff decolonisation occurred in H1 during this intervening period. Upon reappearing in 2014, the outbreak strain had undergone slight modifications which were detectable using conventional molecular epidemiological typing. Specifically, *spa* typing indicated that the *spa* gene had evolved from t186 to t786 (the latter of which is distinguishable from the former by the absence of one repeat unit), while DNA microarray profiling revealed an unusual *hsdS* deletion. It is highly likely that these alterations occurred locally, either while the strain resided *in-vivo* in a H1 HCW or during the strain's spread in the community, prior to reintroduction into the NICU.

Transmission period 2 (TP2) involved the acquisition of the outbreak strain, ST78-MRSA-IVa, by 20 patients and one HCW, over a 35-month period. Interestingly, the MST indicated that the vector from which patient P6 acquired the outbreak strain (during TP1), may have constituted the source of the outbreak at the beginning of TP2 (Figure 1). Furthermore, as observed during TP1, it appeared that more than one vector was involved in the spread of the outbreak strain during TP2. This was evident from the significant extension of three TP2 isolates (P15, P16, and P17, recovered in H1, H2, and H2, respectively) from the main body of the cluster in which isolates with both earlier and later recovery dates resided (Figure 1). In contrast to TP1, however, the TP2 isolates were recovered at intervals of 0–7 months suggesting that some TP2 patients may have had overlapping stays (Table 1). This circumstance may have contributed to the establishment of a more complex transmission chain during this time period. Although HCW screening is not mandatory in Ireland, it is indicated if transmission continues on a unit despite active control measures, if epidemiological aspects of an outbreak or strain are unusual, or if they suggest persistent MRSA carriage by staff (Irish Department of Health, 2013). This was likely the basis upon which HCW screening took place during TP2, the extent of which, is unknown. Importantly, an average pairwise distance of 10.5 (range: 1–21) between a H1 HCW isolate (W19) and the remaining TP2 isolates indicated that this HCW was likely directly involved in transmitting the outbreak strain to patients during this period. Similarly, a difference of one allele between both a H1/H2 (P15 and P16) and H1/H3 (P13 and P12) isolate pair, indicated that the outbreak strain spread to two additional hospitals. In the case of H3, it is highly likely that patient P12 acquired the outbreak strain in H1, before being transferred to H3. Similarly, although no known patient transfers occurred between H1 and H2, it is possible that a carrier who was not represented in the present study (i.e. a patient not screened during routine surveillance) was transferred from H1 to H2, during TP2. This is particularly feasible given the high frequency with which patients are transferred between Irish hospitals. However, as the employment of specialist healthcare staff by different hospitals is not uncommon in Ireland, it remains possible that the movement of staff facilitated the inter-hospital spread of this strain.

Genotypic data from both the present investigation and previously published studies were considered while determining the putative geographic origin(s) of the outbreak strain. Firstly, cgMLST indicated that the outbreak strain shared an ancestral genotype with an isolate recovered in Australia (Figure 2). Furthermore, a 21 kb *blaZ*, and *cadX*-encoding plasmid was detected in all Australian and Irish ST78-MRSA-IVa isolates, while a second cryptic 2 kb plasmid was detected in two Australian and all Irish ST78-MRSA-IVa isolates. Moreover, both of these plasmids were previously sequenced from the same Australian ST78-MRSA-IVa strain. Finally, ST78-MRSA-IVa is generally reported exclusively from Australia and the rate of travel between Australia and Ireland was consistently high in the years preceding the study period (Australian Government Department of Immigration Citizenship, 2011). Considering these points, it is highly likely that the outbreak strain was imported from Australia, where it is commonly known as Western Australia MRSA-2 (Coombs et al., 2012). Interestingly, a 2012 study reported that while ST78-MRSA-IVa was the second most prevalent strain among HCWs in a Western Australian hospital, it was associated exclusively with persistent carriage (Verwer et al., 2012). This suggests, that even without constituting the predominant clone in a hospital setting, ST78-MRSA-IV colonisation may be particularly likely to persist, a phenomenon which may have contributed the continued spread of this strain to H1 NICU patients over the eight-year study period.

A second CC88-MRSA clone, ST88-MRSA-IVa, was also identified in Irish hospitals during the present study. In Ireland, patients with specific HCA-MRSA risk factors generally undergo screening for MRSA (Irish Department of Health, 2013). These guidelines likely formed the basis upon which three ST88-MRSA-IVa isolates were recovered from three different patients during the study period. However, considering both the lack of epidemiological links between these isolates and more importantly, the high number of alleles by which they differed, it was concluded that this strain was introduced into Irish hospitals on three separate occasions (Figure 1). Moreover, the non-neonatal status of these patients further supported the likelihood of their having acquired this strain (generally considered CA) outside of a healthcare setting, prior to admission.

Extensive genotypic, conventional epidemiological and previously published data were all considered while determining the region(s) from which ST88-MRSA-IVa was likely imported into Ireland. Firstly, cgMLST indicated that two Irish ST88-MRSA-IVa isolates, P9, and P25, shared ancestral genotypes with isolates recovered in Egypt and France, respectively (Figure 2). Furthermore, sequence-based plasmid analysis revealed that all Irish and international ST88-MRSA-IVa isolates harboured the same *blaZ* and *cadX*-encoding plasmid, previously sequenced from a Tanzanian MRSA isolate. This suggested that all ST88-MRSA-IVa investigated may have originated in relatively close geographic proximity. Moreover, an Irish ST88-MRSA-IVa isolate harboured an additional plasmid, previously sequenced from a Ghanaian *S. aureus* isolate. Secondly, ST88 MRSA has become increasingly associated with Africa in recent years and France is known to have a large population of African ethnic origin (Schaumburg et al., 2014; https://www.insee.fr/en/statistiques/1283070). Finally, two of the three patients from whom ST88-MRSA-IVa was recovered, had African names, suggesting they may have had family connections to an African country. Considering these points, it was concluded that ST88-MRSA-IVa was likely imported into Ireland from Africa and/or a country with a large population of African ethnic origin.

While WGS and DNA microarray profiling were successfully utilised to achieve the aims of the present study, two significant limitations, which often impede WGS-based studies, were also identified. Firstly, in the absence of universally accepted intra-host strain diversity guidelines, putative transmission events were not identified exhaustively. Notably, however, isolates recovered from twins (who generally share nursing care) on the same day, differed by eight alleles. Furthermore, isolates P10 and P11, which were recovered 3 days apart following a 30-month period in which the outbreak strain was not detected, differed by nine alleles. This suggests that an approximate intra-host strain diversity threshold of nine alleles may be applicable to the present study. Secondly, a lack of detailed epidemiological information limited the certainty with which conclusions could be drawn regarding the intricate dynamics of the H1 NICU outbreak, thus highlighting the importance of strong communicative links between healthcare facilities and research groups. However, despite the lack of detailed epidemiological information and the long period of time over which isolates were recovered, WGS provided robust and precise evidence of the occurrence of a protracted outbreak. Finally, this study highlighted the importance of considering all available epidemiological and genotypic information while selecting the whole-genome analysis approach best suited to the specific data set in question.

The present study revealed the HCW-facilitated spread of an Australian CA-MRSA strain, ST78-MRSA-IVa, in the NICU of an Irish maternity hospital over an eight-year period. Such findings indicate that further consideration of the role of HCWs in the transmission of MRSA in high-dependency units, such as NICUs, may be beneficial. This study also identified multiple introductions of an African CA-MRSA clone, ST88-MRSA-IVa, into Irish hospitals, suggesting that CA-MRSA risk factors should be considered during targeted patient screening. In a broader context, this study highlighted both the significance of travel in the spread of MRSA and the need for well-designed WGS-based studies that include in-depth epidemiological information in order to aid the establishment of data interpretation guidelines and thus, facilitate the real-time application of WGS in a clinical setting.

## Author contributions

AS, DC, and GB conceived the study and provided the required resources. ME and TF performed the practical work. PS performed the *in silico* microarray and plasmid analysis. ME, AS, SM, RE and DC performed the remaining data analysis. ME and AS wrote the manuscript. DC, SM, RE, PS, and GB critically reviewed and edited the manuscript.

### Conflict of interest statement

SM, RE, and PS are employees of Abbott (Alere Technologies GmbH). The remaining authors declare that the research was conducted in the absence of any commercial or financial relationships that could be construed as a potential conflict of interest.
