# Supplementary material for: Intra-Hospital, Inter-Hospital and Intercontinental Spread of ST78 MRSA From Two Neonatal Intensive Care Unit Outbreaks Established Using Whole-Genome Sequencing
Source: Front Microbiol. 2018 Jul 4;9:1485. doi: 10.3389/fmicb.2018.01485 (PMC6039561; doi:10.3389/fmicb.2018.01485)
Supplement: Supplementary file 2 [file Image_1.PDF]

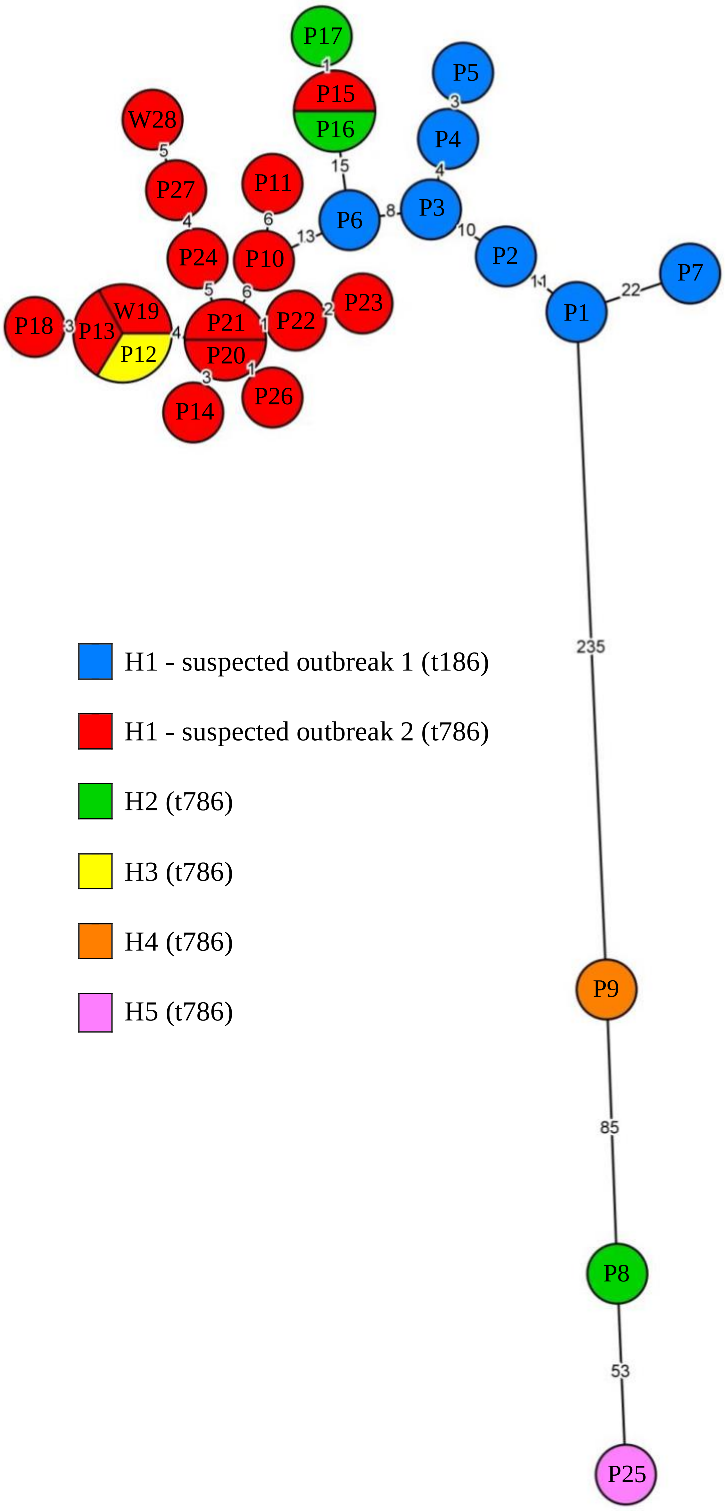

Figure S1 A minimum spanning tree based on core-genome multilocus sequence typing profiles of 28 CC88-MRSA isolates recovered in Irish hospitals between 2009-2017. Isolates are numbered in the order in which they were recovered. Isolate *spa* types are indicated by the colour legend. Branch labels represent allelic distances. All suspected outbreak 1 isolates, both H2 isolates and the H3 isolate exhibited an unusual *hsdS* deletion. The present MST suggests that this deletion occurred twice during the spread of this strain; once in a common ancestor of isolates P6 and P10 and again, in a common ancestor of isolates P6 and P15. However, both whole-genome multilocus sequence typing and single nucleotide polymorphism data suggest that this deletion occurred only once, in a common ancestor of isolates P6 and P10. Abbreviations: H, hospital.

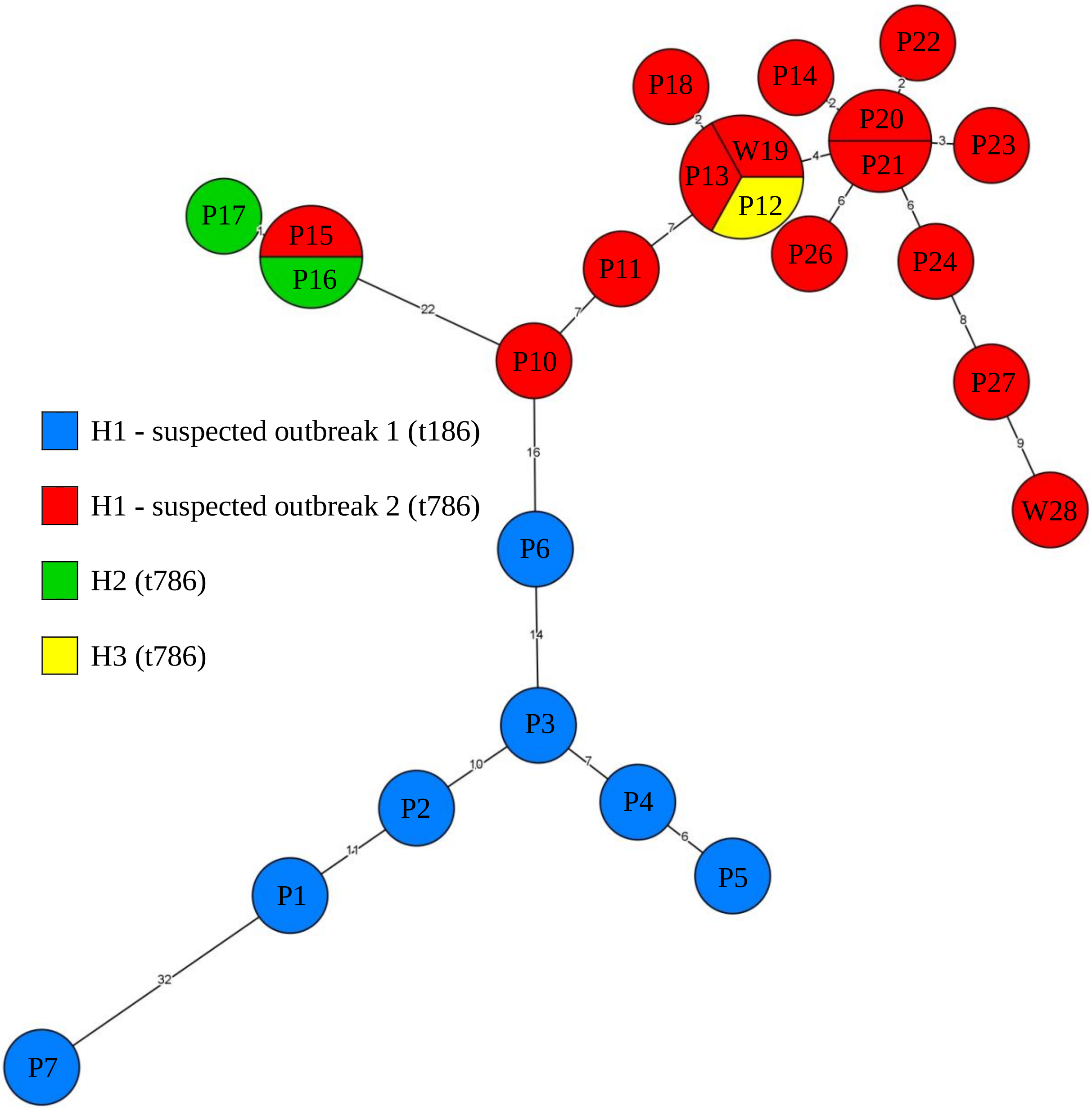

Figure S2 A minimum spanning tree based single nucleotide polymorphism data of 25 CC88-MRSA isolates recovered in Irish hospitals between 2009-2017. Isolates are numbered in the order in which they were recovered. Note the absence of isolates P8, P9 and P25 which were excluded from the present figure due to their distant relationship with the remaining isolates. Isolate *spa* types are indicated by the colour legend. Branch labels represent single nucleotide polymorphism distances. All suspected outbreak 1 isolates, both H2 isolates and the H3 isolate exhibited an unusual *hsdS* deletion. In agreement with whole-genome multilocus sequence typing data, the present figure suggests that this deletion occurred once during the spread of this strain, in a common ancestor of isolates P6 and P10. Abbreviations: H, hospital.

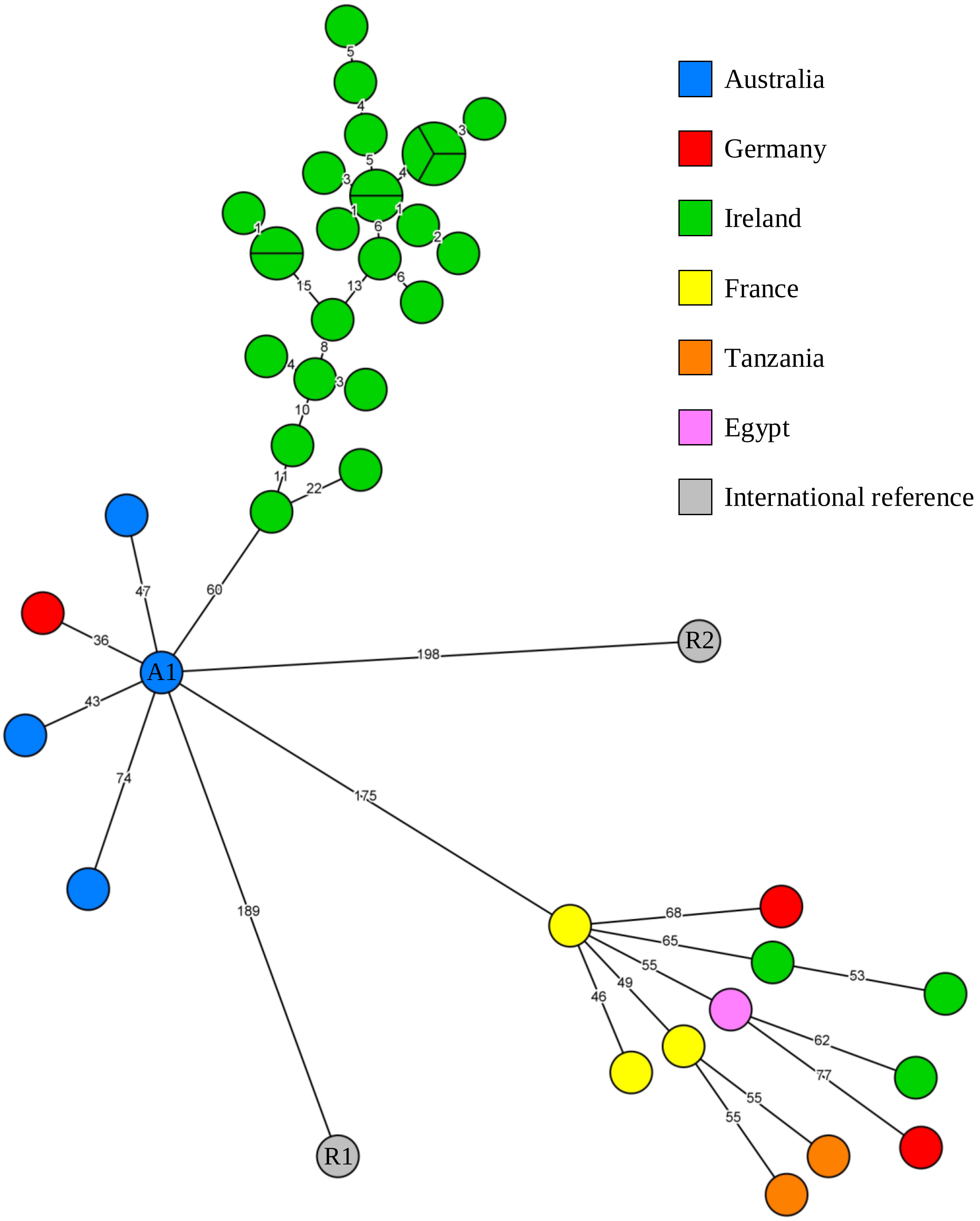

Figure S3 A minimum spanning tree based on core-genome multilocus sequence typing profiles of the 28 Irish CC88-MRSA isolates investigated in the present study and 15 international CC88-MRSA isolates recovered between 2001 and 2017. Thirteen of the international isolates from France, Australia, Germany, Tanzania and Egypt, were selected for comparison to Irish isolates based on the similarity of their hybridisation profiles obtained using the Genotyping Kit 2.0 DNA microarray system (Abbott [Alere Technologies GmbH], Jena, Germany). Two international isolates (R1 and R2) from Germany were selected based on the dissimilarity of their array profiles to the Irish isolates. Branch labels represent allelic distances.
